# Progress in Surgery

**Published:** 1901-08-31

**Authors:** 


### Progress in Surgery.

### GENITO-URINARY SURGERY.

**An Improved Method of Performing Suprapubic Cystotomy.**—C. L. Gibson, New York,[1] has suggested utilising for the urinary bladder the principle which has proved so successful in the creation of a permanent gastric fistula by Kader's method. Gibson considers Kader's operation of gastrostomy the standard one of the present day. A tube is placed in the stomach opening, and the incision reduced to a size that makes it fit snugly. Then a fold of stomach wall is brought around the tube by passing Lembert sutures on either side, and afterwards a second set is similarly applied. The tube is fixed by a stitch passing through it and the stomach wall. In this way a permanent opening is formed in the stomach in a few days, and there is no escape of gastric juice either with the tube *in situ* or after its removal, owing to the inverted cone-shaped valve which projects into the lumen of the stomach. Gibson gives diagrams illustrating the application of similar sutures in suprapubic cystotomy. They show the mode in which two successive rows of Lembert's sutures are applied, each row consisting of four sutures, two above the drainage tube and two below. The weak point of this method when applied to the bladder is the absence of a peritoneal covering such as the stomach possesses. From a limited number of cases Gibson and others have found that the method secures very perfect drainage without leakage. A case of P. R. Bolton's is quoted in which there was no leakage. The tube slipped out accidentally on the third day and remained out several hours, but no leakage occurred. On the fourth day the tube was permanently left out, and from that time not a drop of urine escaped. Again, Bolton Bangs reports that he drained a bladder for 14 days by this method without leakage. On taking out the tube the closure was so prompt that only enough urine to moisten slightly the dressings escaped for the first day or two. The important question remains, whether the method can be utilised, as in gastrostomy, to form a permanent sinus which will allow of continued periodical catheterisation through it with absence of leakage during the intervals. Gibson believes this result can be obtained, but probably not without further elaboration of the present technique. He thinks if less serious measures fail to accomplish this result, he will eventually perform abdominal section, bring the bladder into the wound, and make the fistula through the peritoneal surface. A special double tube permitting irrigation of the bladder may be used instead of the ordinary single one in this operation. No syphon arrangement is needed to get perfect drainage. The ends of the second row of sutures are left long and carried through the parietes. The wound in the latter is closed snugly round the tube.

**Cystoscopy in the Female.**—William R. Pryor, New York,[2] has described an improved method of examining the female bladder, which admits intravesical operations and treatment of the ureters. His instrument consists of a main tube for inspection, to the side of which is attached a smaller tube for carrying the lamp. The illumination tube extends beyond the inspection tube, and therefore no rays of light from the lamp enter the inspection tube directly. The tubes are made of three sizes, the smallest being for use under cocaine. The advantages claimed for this cystoscope are: a tube for inspection free from obstructions and free from light rays either direct or reflected; the absence of necessity for focussing rays of light, which is troublesome when a head-mirror or lamp is used; the passage of the rays of light directly to the object to be inspected; the ease with which demonstrations can be made; absence of heat; absence of urine about the trigone; and absence of the necessity of pumping out urine. Pryor lays great stress upon the position of the patient. If the bladder is examined when it contains liquid a closed tube must be used and catheterisation of the ureters then becomes very difficult. Hence the introduction of the method of distending the bladder with air. Kelly apparently first used a dorsal position with elevated hips. Subsequently he adopted the knee-chest position, which he still uses. In that position often special means, such as distension of the vagina with air or with a cotton plug, are necessary to bring the posterior wall of the bladder forwards out of the hollow of the sacrum and enable the observer to see the ureteral orifices. Pryor has the patient in a position he considers better than the knee-chest one. The patient is placed on her back in the lithotomy position, and has the buttocks elevated considerably above the level of the head and chest. This position is obtained by using a special table which can be inclined to the necessary degree after the patient has been placed upon it. Usually it is lowered at the head end so that the inclination is about 45°. The uterus then sinks away from the pubes, and drags with it that portion of the bladder which is covered with peritoneum, and at the same time straightens it out. The cervix sinks away from the vulva and straightens out the base of the bladder. Thus the bladder acquires the shape of an open equilateral triangle with rounded corners, and the flat surfaces can be more satisfactorily examined than the curved ones which the knee-chest position produces. Pryor remarks upon the excellent demonstration which can be given to students by his method, and says that he has shown both ureters to sixty men and passed ureteral catheters within an hour.

[1] Med. Rec., January 1901. [2] Med. Rec., March 1901.

